# Modelling adaptation strategies to reduce adverse impacts of climate change on maize cropping system in Northeast China

**DOI:** 10.1038/s41598-020-79988-3

**Published:** 2021-01-12

**Authors:** Rong Jiang, Wentian He, Liang He, J. Y. Yang, B. Qian, Wei Zhou, Ping He

**Affiliations:** 1 Ministry of Agriculture Key Laboratory of Plant Nutrition and Fertilizer, Institute of Agricultural Resources and Regional Planning, Chinese Academy of Agricultural Sciences (CAAS), Beijing, 100081 China; 2 Harrow Research and Development Centre, Agriculture and Agri-Food Canada, 2585 County Road, Harrow, ON N0R 1G0 Canada; 3 Institute of Plant Nutrition and Resources, Beijing Academy of Agriculture and Forestry Sciences, Beijing, 100097 China; 4 National Meteorological Centre, Beijing, 100081 China; 5 Ottawa Research and Development Centre, Agriculture and Agri-Food Canada, 960 Carling Ave, Ottawa, ON K1A 0C6 Canada

**Keywords:** Climate-change mitigation, Ecological modelling

## Abstract

Maize (*Zea mays* L.) production in Northeast China is vulnerable to climate change. Thus, exploring future adaptation measures for maize is crucial to developing sustainable agriculture to ensure food security. The current study was undertaken to evaluate the impacts of climate change on maize yield and partial factor productivity of nitrogen (PFPN) and explore potential adaptation strategies in Northeast China. The Decision Support System for Agrotechnology Transfer (DSSAT) model was calibrated and validated using the measurements from nine maize experiments. DSSAT performed well in simulating maize yield, biomass and N uptake for both calibration and validation periods (normalized root mean square error (nRMSE) < 10%, −5% < normalized average relative error (nARE) < 5% and index of agreement (d) > 0.8). Compared to the baseline (1980–2010), the average maize yields and PFPN would decrease by 7.6–32.1% and 3.6–14.0 kg N kg^−1^ respectively under future climate scenarios (2041–2070 and 2071–2100) without adaptation. Optimizing N application rate and timing, establishing rotation system with legumes, adjusting planting dates and breeding long-season cultivars could be effective adaptation strategies to climate change. This study demonstrated that optimizing agronomic crop management practices would assist to make policy development on mitigating the negative impacts of future climate change on maize production.

## Introduction

Maize (*Zea mays* L.) is one of the most important cereal crops to support food security. However, the global average maize yields have declined by approximately 3.8% over the last decades due to climate change^1^. Northeast China is the dominant maize production area in China and plays a unique role in food security. Maize planting area and production in this region account for 31.5% and 32.8% of the total for China, and 6.8% and 7.4% of the global total in 2018, respectively^2,3^. However, Northeast China is one of the most vulnerable regions to climate change, as annual mean temperature has been significantly increasing by 0.38 °C per decade, and precipitation has decreased slightly with more frequent droughts and floods during the last 50 years^4,5^. Previous studies have showed that the negative impacts of climate change on maize yield were mainly associated with the warming and increased drought frequency during the growth periods in Northeast China^6–8^. Maize potential and attainable yields were estimated for a reduction of 2.1% and 8.0%, respectively in Northeast China from 1961 to 2009 due to climate change using the Agricultural Production Systems Simulator (APSIM) model^7^. Maize yield gaps are mainly affected by management practices (e.g., cultivars and fertilizer input) over the period 1961–2010^9^. Thus, adjustment of management practices (e.g., optimizing fertilization and adopting high-yield cultivars) played an important role to close yield gap, improve nutrient use efficiency (NUE) and minimize environmental risks under climate change. Lv et al.^7^ demonstrated that the improved cultivars and agricultural practices could mitigate the negative impacts of climate change from 1961 to 2009 relative to the yields of the cultivar planted in the 1960s. Thus, exploring effective adaptation measures in Northeast China is crucial to improving maize production and maintaining environmental health under future climate change.

Many potential adaptation strategies have been explored to mitigate the negative impacts of maize yield under climate change conditions. Several studies indicated that adjustment of crop phenology could be beneficial for mitigating yield loss under future climate scenarios (e.g., elongating maturity and changing sowing date)^10–12^. For examples, Lin et al.^10^ showed that maize yield loss could be mitigated by substituting local cultivars with later-maturing and delaying the planting date in Heilongjiang province, Northeast China under future climate change. In addition, several other studies also suggested that agronomic practices should be considered as adaptation measures (e.g., optimization of fertilizer application rate and crop rotation system)^13–15^. Fertilizer application rate and timing should be adjusted to meet the nutrient demands of crop growth and avoid nutrient loss when crop biomass decreases due to water and temperature stress over time under climate change^14,15^. For example, He et al.^14^ explored the response of maize yields to fertilizer application rate under future climate scenarios, which suggested that the nitrogen (N) rate of 150 kg N ha^−1^ would be suitable for high maize yields in Canada based on the Decision Support System for Agrotechnology Transfer (DSSAT) model. Crop rotation diversity is beneficial to improving soil physical quality, nutrient availability and soil microbial diversity, which contributes to high crop yield and low environmental risk^16–18^. Ma et al.^19^ predicted that including legumes in rotation would be advocated for mitigation under a changing climate, which would increase crop yields by about 5% for rainfed agricultural systems in Australia. Therefore, exploring adaptation strategies based on comprehensive agronomic management practices are essential to promoting sustainable maize production in Northeast China under future climate change.

Process-based models are valuable tools in evaluating management practices and climate change impacts on crop production, soil water balance and carbon (C) & N dynamics in diverse agroecosystems^20,21^. The DSSAT model is a widely used tool for testing cropping technologies, assessing management practices, and exploring climate change mitigation stategies^14,21–24^. The DSSAT model has been successfully used to optimize field management practices to achieve high crop yield, improve understandings of crop physiology, soil management and weather effects on crop growth and environmental quality^25,26^, and explore the responses of crop production to climate change and develop effective adaptation strategies^10,12,14,27^. Although the DSSAT model has been used to simulate the impacts of climate change on maize yield, no detailed adaptation strategies were assessed in simulations by considering comprehensive management practices for improving maize production and N use efficiency in Northeast China. Therefore, the objectives of this study were (1) to calibrate and evaluate the DSSAT model using the measured maize yield, biomass and N uptake from nine field experiments in Northeast China; (2) to simulate the climate change impacts on maize yield and partial factor productivity of N (PFPN) during two future periods of 2041–2070 and 2071–2100, relative to the baseline (1981–2010) under Representative Concentration Pathways (RCP) 4.5 and 8.5 scenarios; and (3) to explore potential adaptation strategies to reduce the negative impacts of future climate change on maize yield and N use efficiency.

## Results

### Model calibration and validation

For calibration, the simulated maize yield, biomass and plant N uptake matched well with the measured data under the optimum nutrient application (OPT) treatment at all sites. The average statistical values of nRMSE ≤ 6.0%, −4.8% ≤ nARE ≤ 0.4% and d ≥ 0.79 indicated “good” to “excellent” agreements between the simulated and measured maize yield, biomass and plant N uptake (Table 1 and see Supplementary Fig. S1, 2). For validation, the model performance was “good” to “excellent” for maize yield, biomass and N uptake simulation under the farmers’ practice (FP) treatment at all sites based on the average statistical value of nRMSE ≤ 6.4%, −1.4% ≤ nARE ≤ 3.3% and d ≥ 0.76 (Table 1 and Supplementary Fig. S1, 2). Additionally, the model slightly overestimated maize yield and N uptake for the FP treatment at all sites, except for the maize yield at HLJ site. Overall, simulations were in agreement with the observations under both the OPT and FP treatments across all sites.

**Table 1:** Statistical evaluation between the simulated and measured maize yields, biomass and plant nitrogen (N) uptake from 2011 to 2016 at Liaoning (LN), Jilin (JL) and Heilongjiang (HLJ) provinces in Northeast China.

	Item	Calibration (OPT)			Validation (FP)		
		LN	JL	HLJ	LN	JL	HLJ
Maize yield	Measured (Mg ha^−1^)	10.20	10.09	9.36	9.88	9.74	9.09
	Simulated (Mg ha^−1^)	10.15	9.92	9.35	10.19	9.85	8.96
	nRMSE (%)	4.7	4.0	5.0	5.3	4.7	6.2
	nARE (%)	− 0.5	− 0.8	− 0.1	3.2	0.9	− 1.4
	d	0.79	0.93	0.85	0.78	0.91	0.83
Biomass	Measured (Mg ha^−1^)	20.71	19.83	20.70	20.45	19.97	19.55
	Simulated (Mg ha^−1^)	20.63	19.74	20.47	20.71	19.93	19.77
	nRMSE (%)	3.1	3.4	4.3	3.6	3.8	4.8
	nARE (%)	− 0.3	0.0	− 1.1	1.3	0.1	1.1
	d	0.87	0.86	0.86	0.84	0.86	0.88
Plant N uptake	Measured (kg N ha^−1^)	212	184	185	216	187	176
	Simulated (kg N ha^−1^)	202	183	185	223	195	177
	nRMSE (%)	6.0	4.0	5.9	5.1	6.2	6.4
	nARE (%)	− 4.8	0.1	0.4	3.3	3.2	1.0
	d	0.80	0.83	0.87	0.79	0.76	0.82

*OPT* optimum nutrient application, *FP* farmers’ practice, *nRMSE* normalized root mean square error, *nARE* normalized average relative error, *d* index of agreement.

### Climate change impacts on maize yield and N use efficiency

**Figure 1:**
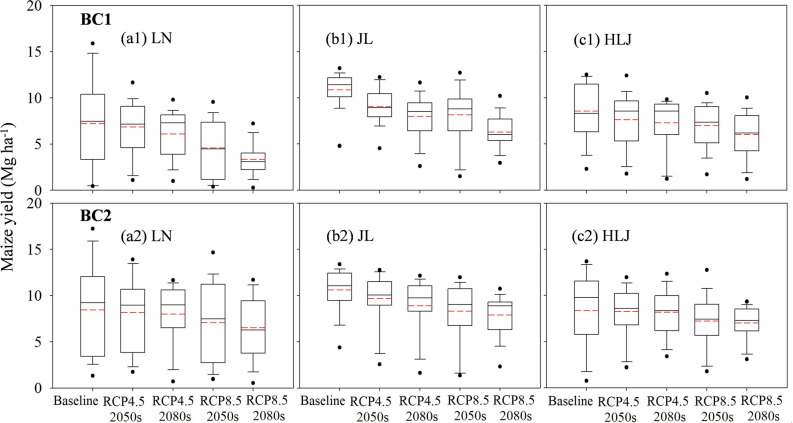
Effects of climate change scenarios on maize yields under BC1 (**a1**, **b1**, **c1**) and BC2 (**a2**, **b2**, **c2**) climate scenarios at Liaoning (LN), Jilin (JL) and Heilongjiang (HLJ) provinces in Northeast China. The black (solid) and red (dashed) lines, lower and upper edges of the boxes, and bars and dots outside the boxes represent median and mean values, 25th and 75th, 5th and 95th, and < 5th and > 95th percentiles of all data, respectively.

The maize yields were simulated using the DSSAT model for the baseline (1980–2010) and two future periods [2050s (2041–2070) and 2080s (2071–2100)] based on two climate change scenarios (RCP4.5 and RCP8.5) (Fig. 1). The maize yields in climate model BCC-CSM1.1 (m) (BC2) were higher than those of in BCC-CSM1.1 (BC1), except at JL and HLJ sites under the baseline scenario. Compared to the baseline, the average maize yields of BC1 and BC2 decreased by 4.4, 10.8, 27.7 and 40.2% for LN, 12.6, 21.1, 23.1 and 33.6% for JL, and 5.9, 8.4, 15.6 and 22.6% for HLJ under the RCP 4.5 2050s, RCP 4.5 2080s, RCP 8.5 2050s and RCP 8.5 2080s, respectively. Under the RCP 4.5 scenarios, the greatest negative impacts of climate change on maize yields and PFPN were observed at JL sites, followed by LN and HLJ. Under the RCP 8.5 scenarios, however, the descending order of the negative impacts changed to LN, JL and HLJ. The average PFPN ranged from 35.7 to 23.4 kg N kg^−1^, 44.6 to 33.7 kg N kg^−1^ and 44.2 to 36.2 kg N kg^−1^ at LN, JL and HLJ respectively under future climate scenarios (Supplementary Fig. S3).

### Potential adaptation measures under future climate scenarios

**Figure 2:**
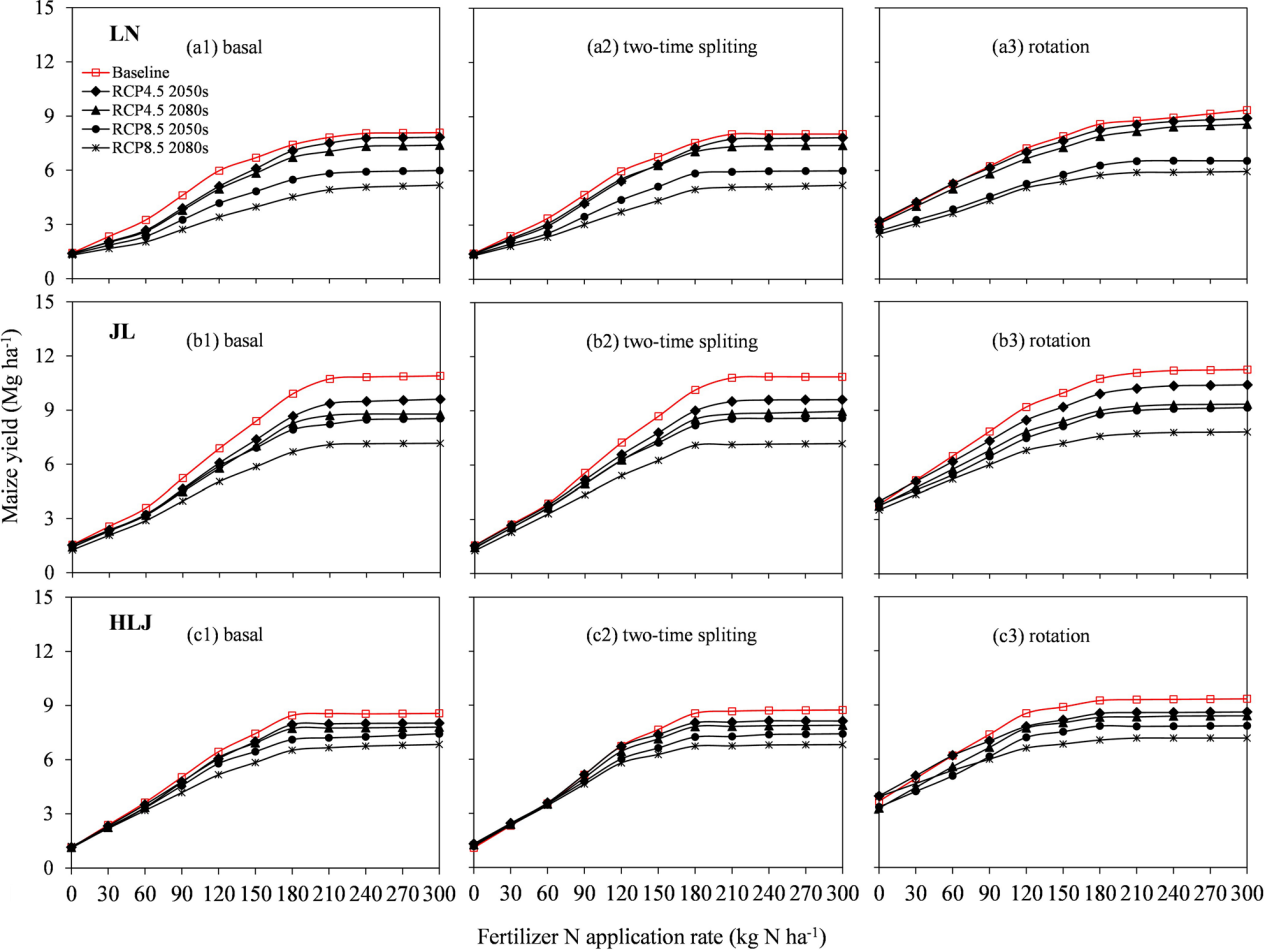
Responses of maize yields to nitrogen (N) application rate as basal (**a1**, **b1**, **c1**), as two-time splitting (**a2**, **b2**, **c2**) for maize monoculture and as basal for maize-soybean rotation (**a3**, **b3**, **c3**) under climate change scenario BC1 (BCC-CSM1.1) at Liaoning (LN), Jilin (JL) and Heilongjiang (HLJ) provinces in Northeast China.

Maize yield response to N application rate and timing. The simulated maize yields dramatically increased with an increase of N application rate for all scenarios, and maize yields leveled off when the N application rate exceeded 240 kg N ha^−1^ at LN and JL sites, and 210 kg N ha^−1^ at HLJ (Fig. 2 and Supplementary Fig. S4). Compared to the 210 and 240 kg N ha^−1^ as basal fertilizer, higher or comparable maize yields were observed at 180 kg N ha^−1^ as two-time splitting at HLJ and at 210 kg N ha^−1^ as two-time splitting at LN and JL respectively under all climate scenarios. Compared to the default values, the average maize yields increased by 2.7%, 0.9% and 1.1% at LN, JL and HLJ when the N application rate at 210, 210 and 180 kg N ha^−1^ as two-time splitting under baseline scenario, while the average maize yields increased by 2.1–4.2%, 0.5–4.6% and 1.0–3.2% under future RCP scenarios, respectively (Table 2). Compared to the default N management, the average PFPN slightly increased by 0.6–1.4 kg N kg^−1^ at LN, by 0.2–1.9 kg N kg^−1^ at JL, and by 0.4–1.2 kg N kg^−1^ at HLJ under future climate scenarios based on the optimized N application rates and timing (Supplementary Table S1). The results indicated that the N application rates of 180–210 kg N ha^−1^ as two-time splitting at LN and JL, and 150–180 kg N ha^−1^ at HLJ are appropriate to achieve a stable and higher yield for both current and future climate change conditions.

**Table 2:** Potential adaptation management practices for maize yields under climate change scenarios at Liaoning (LN), Jilin (JL) and Heilongjiang (HLJ) provinces in Northeast China.

Site	Management	Item	Optimized	Maize yield (Mg ha^−1^)				
				Baseline	RCP 4.5 2050s	RCP 4.5 2080s	RCP 8.5 2050s	RCP 8.5 2080s
LN	Default	–	–	7.8	7.5	7.0	5.8	4.9
	N rate (monoculture)	Base	240	8.0 (2.9%)	7.8 (3.6%)	7.3 (4.0%)	5.9 (1.7%)	5.1 (2.9%)
	(kg N ha^−1^)	Base and side-dress	210 (1/3 and 2/3)	8.0 (2.7%)	7.8 (3.3%)	7.3 (4.2%)	5.9 (2.1%)	5.1 (3.4%)
	N rate (rotation) (kg N ha^−1^)	Base	210	8.8 (12.0%)	8.5 (13.7%)	8.2 (16.0%)	6.5 (12.0%)	5.9 (19.8%)
	Planting date (day of year)	Baseline/future	133/143	8.4 (7.2%)	8.5 (13.8%)	7.9 (12.8%)	6.8 (16.7%)	6.1 (24.0%)
	Cultivar parameters (°C.d.)	P1	325–375	8.0 (2.5%)	7.7 (3.2%)	7.5 (6.6%)	6.4 (10.6%)	5.2 (5.6%)
		P5	980	8.6 (10.3%)	8.6 (14.0%)	8.2 (15.8%)	6.8 (16.4%)	5.7 (16.3%)
JL	Default	–	–	10.7	9.4	8.4	8.2	7.1
	N rate (monoculture)	Base	240	10.8 (1.0%)	9.6 (2.3%)	8.8 (4.0%)	8.5 (3.0%)	7.1 (0.7%)
	(kg N ha^−1^)	Base and side-dress	210 (1/3 and 2/3)	10.8 (0.9%)	9.5 (1.7%)	8.8 (4.6%)	8.5 (4.0%)	7.1 (0.5%)
	N rate (rotation) (kg N ha^−1^)	Base	210	11.1 (3.1%)	10.2 (9.0%)	9.2 (9.2%)	9.0 (9.3%)	7.7 (8.9%)
	Planting date (day of year)	Baseline/future	113/143	10.7 (0.1%)	9.5 (1.5%)	9.2 (9.2%)	8.9 (7.9%)	8.0 (12.3%)
	Cultivar parameters (°C.d.)	P1	260–290	10.7 (0%)	9.7 (3.7%)	9.1 (7.8%)	8.8 (6.9%)	7.4 (4.4%)
		P5	920	11.6 (8.3%)	11.1 (18.5%)	10.0 (19.0%)	9.8 (19.3%)	8.4 (18.2%)
HLJ	Default	–	–	8.5	7.9	7.7	7.1	6.5
	N rate (monoculture)	Base	210	8.6 (1.2%)	8.0 (0.4%)	7.8 (0.3%)	7.2 (1.4%)	6.7 (2.2%)
	(kg N ha^−1^)	Base and side-dress	180 (1/3 and 2/3)	8.5 (1.1%)	8.0 (1.0%)	7.8 (1.0%)	7.2 (1.7%)	6.7 (3.2%)
	N rate (rotation) (kg N ha^−1^)	Base	180	9.2 (9.4%)	8.5 (7.3%)	8.3 (7.5%)	7.8 (10.1%)	7.1 (8.2%)
	Planting date (day of year)	Baseline/future	138/148	8.9 (5.5%)	9.5 (19.6%)	9.1 (18.3%)	8.6 (21.2%)	7.8 (19.1%)
	Cultivar parameters (°C.d.)	P1	210–235	8.5 (0%)	8.4 (5.2%)	8.2 (5.8%)	7.7 (7.9%)	7.1 (8.4%)
		P5	856	9.4 (11.1%)	10.0 (25.3%)	9.9 (27.5%)	8.9 (24.9%)	8.3 (27.5%)

The content within the brackets represents the change of potential adaptation management practices on maize yields compared to the default. P1, Thermal time from seedling emergence to the end of the juvenile phase (degree days > 8 °C); P5, Thermal time from silking to physiological maturity (degree days > 8 °C).

#### Maize yield response to maize-soybean rotation

Compared to maize monoculture, higher maize yields were obtained with maize-soybean (*Glycine max* [L.] Merr) rotation under both baseline and future climate scenarios (Fig. 2 and Supplementary Fig. S4). The average maize yields from maize-soybean rotation increased by 12.0–19.8%, 3.1–9.3%, and 7.3–10.1% across all scenarios at LN, JL and at HLJ, respectively, compared to the default maize cropping system (Table 2). Particularly, similar maize yields could be achieved when the N application rate was 150–180 kg N ha^−1^ as basal at LN and JL, and 120–150 kg N ha^−1^ as basal at HLJ compared to that of 180–210 kg N ha^−1^ and 150–180 kg N ha^−1^ as two-splitting for maize monoculture under future climate scenarios, respectively. The average PFPN was increased by 3.3–5.4, 1.6–4.0 and 3.2–4.0 kg kg^−1^ at LN, JL and HLJ respectively, compared to the default values for all scenarios (Supplementary Table S1).

#### Maize yield response to planting date

**Figure 3:**
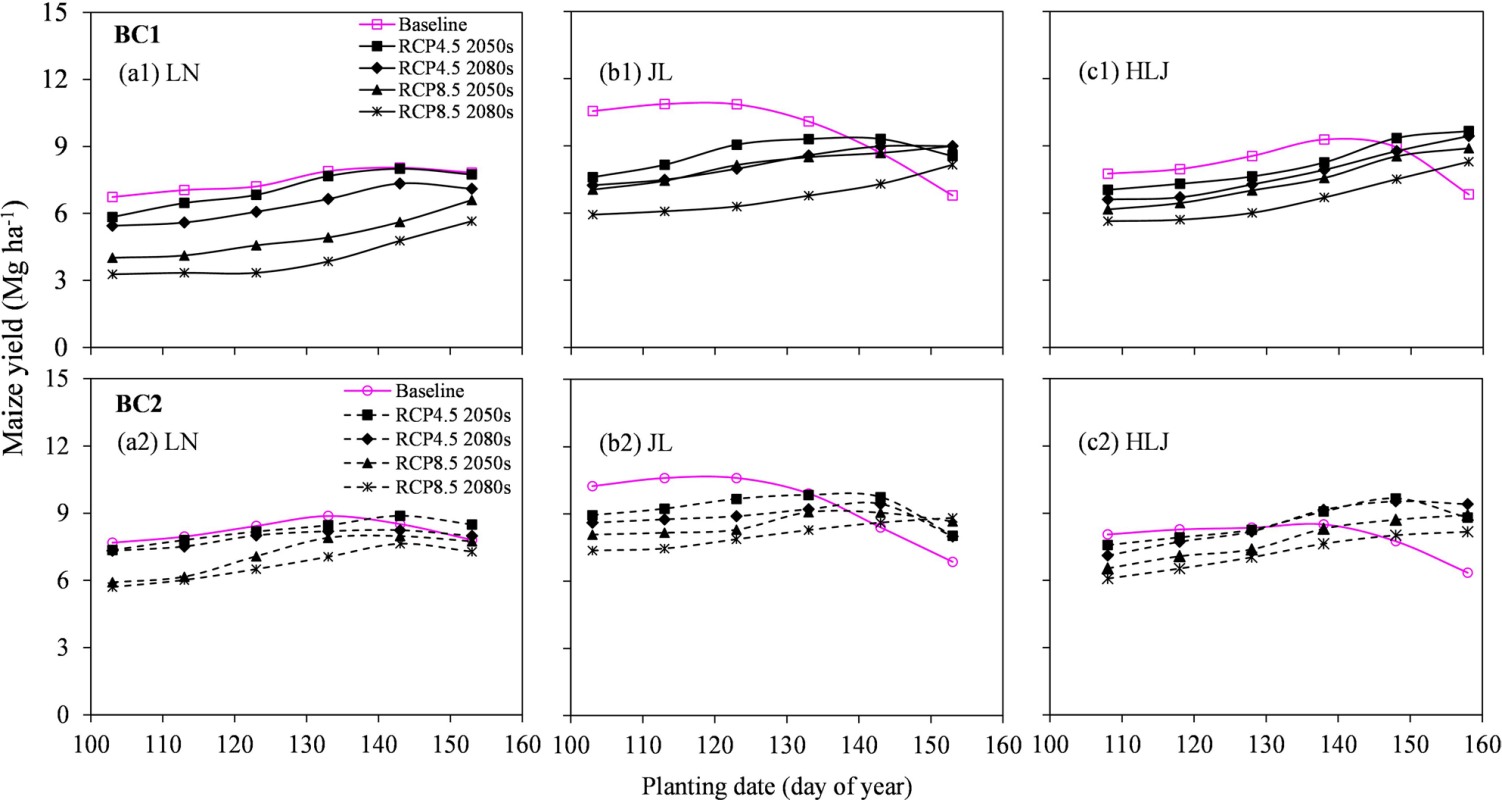
Responses of maize yields to planting date under climate change scenarios BC1 (**a1**, **b1**, **c1**) and BC2 (**a2**, **b2**, **c2**) at Liaoning (LN), Jilin (JL) and Heilongjiang (HLJ) provinces in Northeast China.

Changing planting date significantly affected the maize yields under both baseline and future climate change scenarios at all sites (Fig. 3). Under baseline scenario, the optimal planting dates (Julian day) ranged from 123 to 133 at LN, 113 to 123 at JL and 128 to 138 at HLJ. However, the maize yield significantly decreased by 6.8% when the planting dates were delayed by 10 days compared to the current seeding dates (123 day) at JL, which was likely attributed to the insufficient thermal time to maturity. In contrast, the negative impacts of future climate scenarios on maize production would be mitigated by delaying the planting 10–20 days (Table 2 and Fig. 3). For example, the average maize yields would increase by 5.9–30.1% for LN, 1.8–12.7% for JL and 9.0–19.8% for HLJ with increased PFPN if the planting dates were delayed to 133–143 (at LN and JL) and 138–148 (at HLJ) compared to the default date under future RCP scenarios (Fig. 3 and Supplementary Table S1). For future climate scenarios, late planting was conducive for increasing maize yields and PFPN at all sites.

#### Maize yield response to cultivar parameters

**Figure 4:**
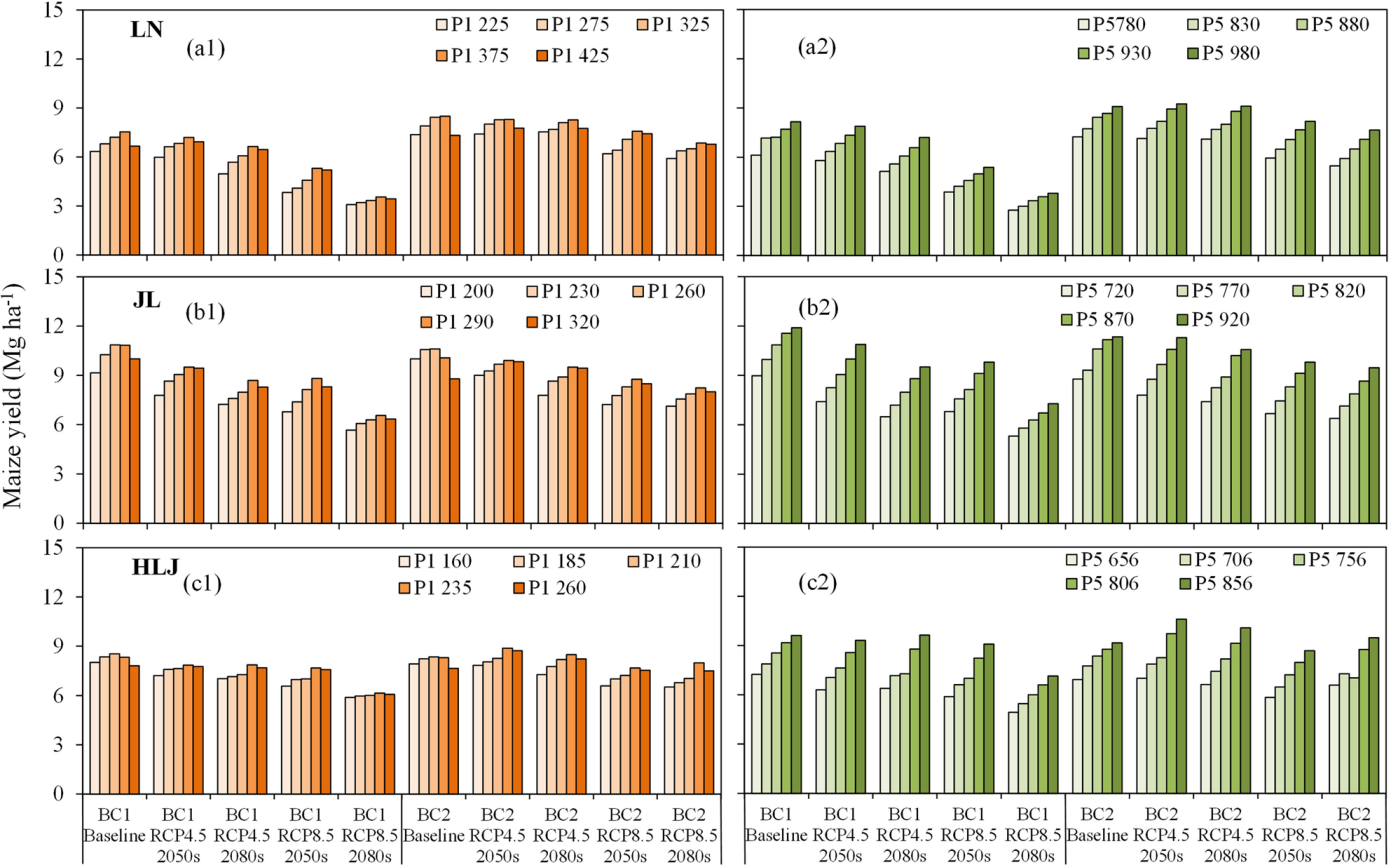
Responses of maize yields to cultivar parameters P1 (**a1**, **b1**, **c1**) and P5 (**a2**, **b2**, **c2**) under climate change scenarios at Liaoning (LN), Jilin (JL) and Heilongjiang (HLJ) provinces in Northeast China.

As shown in Fig. 4, the average maize yields would increase by 3.2, 6.6, 10.6 and 5.6% when P1 changed from 320 (default) to 375 under RCP 4.5 2050s, RCP 4.5 2080s, RCP 8.5 2050s and RCP 8.5 2080s at LN, while the maize yields decreased when P1 exceeded 425 under baseline scenario, which was mainly due to the long thermal time from seeding to juvenile causing insufficient thermal time for maturity. Similarly, the average maize yields would decrease when P1 exceeded 290 and 235 at JL and HLJ, respectively. The average maize yields would significantly increase when changing P5 parameters from the default values (880, 820 and 756) to 980, 920 and 856 degree days at LN, JL and HLJ under all future climate scenarios, respectively (Table 2 and Fig. 4). In addition, the average PFPN showed similar trends with maize yield when changing the values of P1 and P5 at each experimental site (Supplementary Table S1).

## Discussion

The DSSAT model provided reasonable prediction of grain yields, biomass and N uptake for maize cropping system under various management practices and soil conditions across all sites in Northeast China. The slight overestimation of N uptake under the FP treatments was partially related to the model overestimating N mineralization under overused fertilizer application as basal. A similar result has been reported by Liu et al.^28^, who showed that the over-prediction of the N uptake of maize might be due to an overestimation of N mineralization under sufficient N supply condition.

In this study, future climate scenarios would have negative impacts on maize yields in Northeast China based on the DSSAT simulation, which was consistent with other modelling studies^10,29^. The significant decline of maize yields under future climate scenarios could be related to the increased temperature. The higher temperature in growing season under the RCP8.5 scenarios (Table S2) showed significant negative impacts on maize yields due to the increased heat stress compared to the RCP4.5 scenarios across all sites, especially at LN sites, where the temperature reached 30 °C at the flowering stage. This stage can be shortened by heat stress as it is most sensitive to high temperature, resulting in reduced crop yield^30^. In our study, maize yields were also affected by water stress, thus the increased frequency of dry years in future scenarios would have significant negative impacts on rainfed maize production. Guo et al.^8^ reported that maize yield reduced by a range from 1.6 to 2.7% in Northeast China under RCP4.5 scenarios due to drought during the sensitive phases of the crop, mainly at the milky-mature and sowing-jointing. Although the increased solar radiation was found under the future scenarios (Table S2), the abundant solar and thermal resources after silking were not efficiently utilized for maize yield due to heat stress for spring maize^31^. Liu et al.^32^ showed that an increase in maximum temperature could reduce maize yield during 1981–2010 in the same region when the APSIM model was used. Additionally, a previous study indicated the elevated CO_2_ concentration resulted in a slight increase in maize production which was partially offset by the opposite impacts of increased temperature^33^. The smaller positive effect from elevated CO_2_ concentration was simulated partially due to the fact that maize is a C4 crop with minor impacts of CO_2_ fertilization on C assimilation^34^. Hatfield et al.^35^ indicated that the increased CO_2_ concentration showed less than 10% positive effects on C4 crop.

It has been reported that the negative impacts of climate change on crop productivity could be mitigated by effective agronomic adaptation techniques and seed genetic improvement^9,14,15,19^. Previous modelling studies demonstrated that adjustment of fertilizer over time should be considered in climate change assessment, especially when climatic factors have obvious influence on crop production^13–15^. Our simulation showed that the appropriate fertilizer application rate and timing could result in higher maize yields and PFPN for both baseline and future climate scenarios, but excessive nitrogen application with low nutrient use efficiency (Table S1) led to resources waste and environmental pollution (e.g., greenhouse gas emission and nitrate leaching)^36^. N fertilizer with splitting application could improve the temporal synchronicity between crop N demand and soil N availability, thereby increasing crop yield and reducing residual soil nitrogen and environment risk^37,38^. The optimal N application rates were reduced under the RCP8.5 scenarios at LN and JL sties because water and temperature stresses were the dominant factors on maize growth compared to N stress under climate change. The accuracy of adjustment of fertilizer rate should be based on changing soil organic carbon (SOC) mineralization and crop needs^15^. Crop rotation diversity is an important management practice to increase crop yield and reduce environment pollution by modifying the soil environment across a wide range of soil types and climatic conditions^16–18^. Smith et al.^15^ predicted that yields increased for rotational maize under climate change due to higher SOC and reduced crop water stress in Canada, meanwhile, the lower simulated N runoff loss was found in rotational maize and higher nitrous oxide (N_2_O) emissions, but the lower annual N_2_O emissions were observed during the entire rotation. Our simulation showed that the negative impacts of future climate change on maize yields could be mitigated under the maize-soybean rotation system mainly due to the improved soil fertility, soil water utilization and nutrient use efficiency^39–41^. Previous study indicated that maize yield had stronger responses with legume rotation than other cereal crops (e.g., millet and sorghum)^39^.

Changing planting date is considered an effective adaptive strategy to mitigate the negative impacts of climate change on crop yields on a global scale, such as in Northeast China for maize^10^, in Burkina Faso for maize^11^, in the west and northwest Iran for wheat^12^ and in Canada for spring wheat and maize^14^. These adverse impacts of climate change could be partially offset by optimal planting date, mainly due to the ability to match crop growth with changed temperature and rainfall distribution. Our results indicated that delaying planting date would be beneficial for maize yield under future climate change across all sites (Fig. 3). This is mainly being attributed to the increased precipitation during late July to early August, which resulted in higher pollination rates and kernel numbers without water stress^31^. Suitable increase in temperature and abundant solar radiation would be beneficial to germination of maize seeds^42–44^. Additionally, thermal time from seedling emergence to the end of the juvenile phase (P1) and thermal time from silking to physiological maturity (P5) are the most sensitive periods for maize yields^21^. In the CSM-CERES-Maize model, the cumulative thermal time from emergence to tassel initiation and daily development rate of maize from silking to maturity could be calculated based on P1 and P5 parameters^21,45^. Reasonable increase of the values of the parameters P1 and P5 would mitigate the negative impacts of future climate change on maize yields based on the DSSAT simulation, implying that longer growing season cultivars should be developed in order to benefit the maize crop from longer growing seasons under warmer future climates and to cope with heat stress. Our study was in agreement with He et al*.*^14^ who indicated that breeding longer growing season cultivars with high thermal degree days could result in higher maize yield under future climate scenarios based on modelling using the DSSAT model.

Overall, changes in simulated maize yield under future scenarios could be explained based on crop-climate-soil interactions. However, the uncertainties in projections of climate change impacts on crop growth in agro-ecosystems are large and unavoidable as they inherited from uncertainties in climate and crop models, accuracy in model calibration and evaluation, and change in socio-economic emission scenarios^46,47^. In this study, the dynamic measurements of soil inorganic N (NO_3_^−^–N and NH_4_^+^–N) were not available to calibrate and evaluate soil N simulations during the field experimental periods, which may cause uncertainty in long-term C & N feedback under climate change scenarios. In the model simulation, soil parameters were assumed to remain constant in the future, but certain soil physical properties (e.g., hydraulic conductivity, water holding capacity) may change under different management practices (e.g., fertilizer, tillage, rotation) which could further affect maize yield and soil process^14,25^. Basso et al.^48^ and Smith et al.^15^ indicated that soil C & N dynamics and water status could be affected if soil physical properties were re-initialized in a long-term simulation. In addition, most crop models including the DSSAT model do not incorporate the explicit simulation of the heat impacts on male and female flowering, fertilization of female flowers, and kernel abortion which may lead to more uncertainties in predicting crop production under future climate scenarios^49^. Furthermore, although the DSSAT model performed well in simulating crop yield and nutrient cycling under various soil and crop management practices, the model does not simulate the direct impacts of pests and/or diseases, extreme weather (e.g., flooding, hails and damaging winds) and complex nutrient transfer processes^50^, which could lead to the uncertainties in the simulation.

## Conclusions

The well calibrated DSSAT model was proven a capable tool for assessing climate change impacts on maize yield and NUE and exploring adaptation strategies under RCP 4.5 and RCP 8.5 scenarios in Northeast China. Our study indicated that maize yields and NUE would significantly decrease under future climate scenarios at LN, JL and HLJ provinces compared to the baseline scenario if no adaptation measures were taken in the simulations. Optimized fertilization rate at 180–210 kg ha^−1^ with two time-splitting at LN and JL and at 150–180 kg ha^−1^ at HLJ would result in high maize yields and NUE under future climate scenarios. Maize-soybean rotation with lower fertilizer application rates could be beneficial to obtaining higher maize yields compared to maize monoculture for both baseline and future climate scenarios. Late planting could mitigate the negative impacts of climate change on maize partially due to the increased precipitation in July and August in the future. Developing longer growing-season cultivars should be recommended to obtain stable and high maize yield under future climate change conditions. Optimized agronomic crop management practices could be considered as effective adaptation strategies to climate change for maize production in Northeast China.

## Materials and methods

### Experimental sites

**Figure 5:**
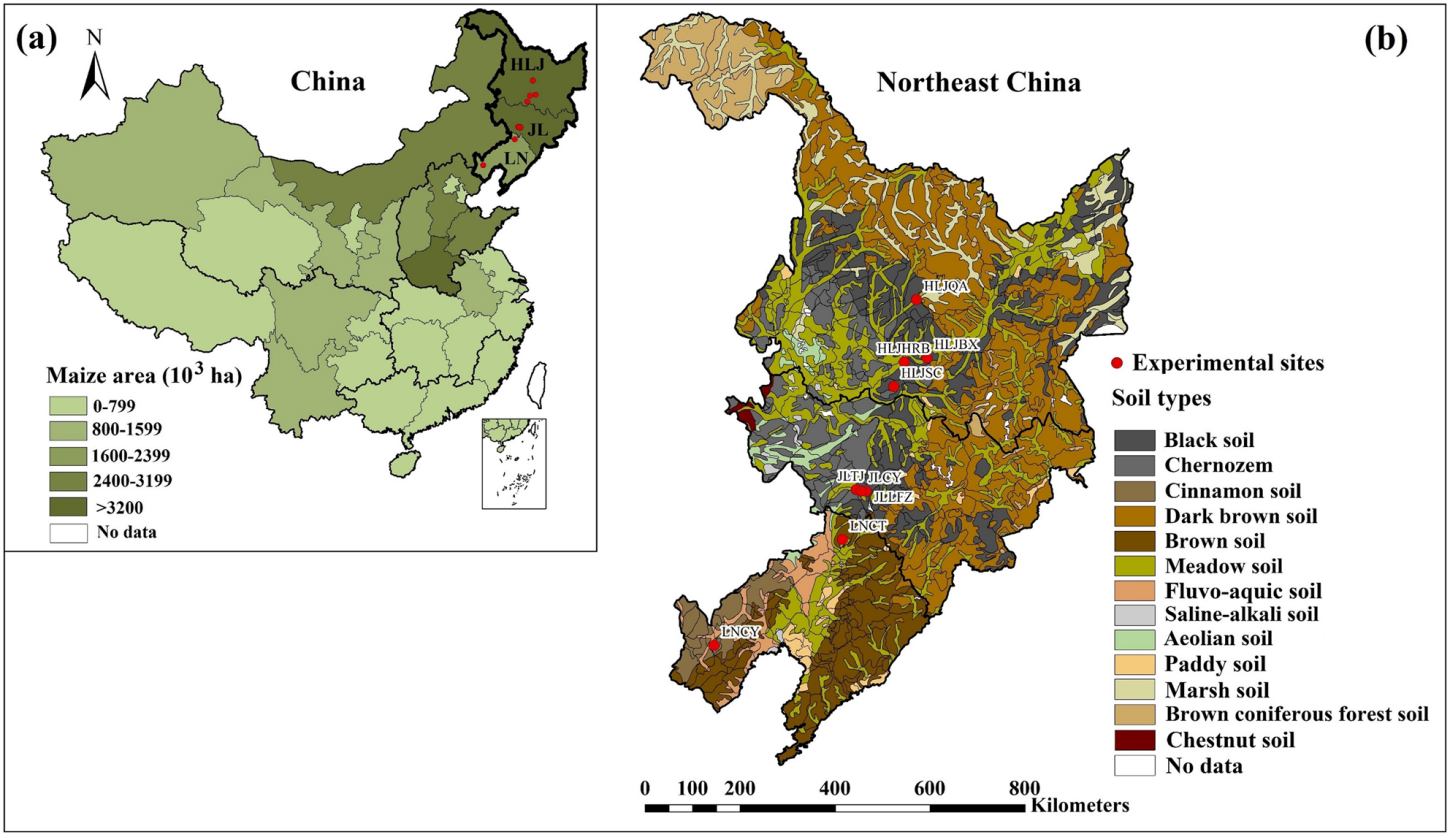
Field experimental sites for maize from 2011 to 2016 at Liaoning (LN), Jilin (JL) and Heilongjiang (HLJ) provinces in Northeast China. The map was created using ArcGIS software 10.4.1 (Environmental Systems Research Institute Inc., Redlands, USA). The area of maize (**a**) was obtained from the National Bureau of Statistics of China (http://data.stats.gov.cn) and soil type information (**b**) was available from the Soil Science Database (http://vdb3.soil.csdb.cn).

Field experiments were conducted from 2011 to 2016 in Northeast China, where is the main maize production region comprising of Liaoning (LN), Jilin (JL) and Heilongjiang (HLJ) provinces. There were nine experimental sites including Chaoyang (LNCY) and Changtu (LNCT) in LN; Liufangzi (JLLFZ), Taojia (JLTJ) and Chaoyang (JLCY) in JL; and Qiangan (HLJQA), Shuangcheng (HLJSC), Binxian (HLJBX) and Harbin (HLJHRB) in HLJ (Table 3 and Fig. 5). In this study, the experimental sites under rainfed conditions are from the main maize planting counties in each province which accounted for 13.6%, 19.1% and 22.5% of the total maize area for LN, JL and HLJ provinces, respectively based on the average values from 2011 to 2016^51–53^. The typical maize cultivars at these sites were selected to represent regional differences of the climatic conditions. These experimental sites were initially established to optimize fertilization management with high quality datasets including maize yield, biomass, nutrient uptake and soil properties which provided detailed inputs to run the DSSAT model. Thus, using modelling approach in major maize planting areas with typical cultivars is feasible to explore potential adaptation management practices under future climate change in Northeast China. Detailed field experimental practices for each site are shown in Table 3. The average air temperature and precipitation during the maize growing season (May to September) were 21.4 °C and 462 mm for LN, 20.5 °C and 527 mm for JL, and 19.5 °C and 503 mm for HLJ (Supplementary Table S3). The basal soil physical and chemical properties at the beginning of the experiments are listed in Table 3.

**Table 3:** Field management practices and soil properties (0–0.2 m) for maize study sites from 2011 to 2016 at Liaoning (LN), Jilin (JL) and Heilongjiang (HLJ) provinces in Northeast China.

Site	Liaoning		Jilin			Heilongjiang			
	LNCY	LNCT	JLCY	JLTJ	JLLFZ	HLJQA	HLJSC	HLJBX	HLJHRB
**Location**									
Latitude (°N)	41.28	42.78	43.61	43.66	43.58	46.96	45.43	45.80	45.84
Longitude (°E)	120.05	123.96	124.76	124.64	124.90	127.68	126.37	127.49	126.85
Altitude (m)	317	145	175	239	229	183	178	167	118
Climate type	Semi-humid	Semi-humid	Humid	Humid	Humid	Semi-humid	Semi-humid	Semi-humid	Semi-humid
**Field management**									
Period	2011–2012	2011–2012	2012–2014	2012–2016	2012–2016	2012–2014	2012–2016	2012–2016	2012–2016
Cultivar	FY9	TY120	YH33	ZF62	NH101	MJN205	LM33	HN1	GF1
Type of maturity	Late	Mid-late	Late	Mid-late	Mid-late	Mid-late	Mid-late	Mid-late	Late
Planting date (day of year)	118–129	106-111	119–121	121–129	119–125	124–139	112–131	117–136	117–138
Harvest date (day of year)	273–278	266-271	272–274	271–274	272–273	272–282	270–282	269–277	271–285
Planting density (seed m^−2^)	5.4–6.0	5.0–6.0	5.8–6.0	5.7–6.5	5.0–6.5	5.5–6.5	5.0–7.5	6.0	5.5–7.0
N application (kg N ha^−1^)	154–211 (234–260)	154–195 (207–240)	150–182 (240–270)	150–211 (208–280)	150–203 (207–280)	154–208 (153)	176–208 (165–198)	167–194 (191–238)	176–182 (170–195)
**Soil property**									
Soil type	Cinnamon soil	Brown soil	Black soil	Black soil	Black soil	Black soil	Chernozem	Black soil	Black soil
pH	8.08	5.30	5.19	5.25	4.92	5.36	5.82	5.32	6.80
Bulk density (g cm^−3^)	1.27	1.32	1.15	1.12	1.18	1.22	1.16	1.21	1.24
Organic carbon (%)	0.65	0.68	0.84	1.77	0.93	2.43	1.49	2.05	2.03
Total nitrogen (%)	0.08	0.09	0.09	0.17	0.12	0.28	0.16	0.22	0.23
Field capacity (m^−3^ m^−3^)	0.280	0.282	0.314	0.321	0.316	0.326	0.320	0.352	0.322
Wilting point (m^−3^ m^−3^)	0.153	0.143	0.152	0.167	0.158	0.166	0.177	0.204	0.180
Saturation (m^−3^ m^−3^)	0.483	0.473	0.498	0.502	0.499	0.504	0.500	0.513	0.502
Clay content (%)	26.6	24.0	28.2	28.3	28.3	29.6	31.7	36.8	34.3
Silt content (%)	29.7	26.5	46.3	46.4	46.3	42.9	33.1	35.3	34.2

LNCY, Liaoning-Chaoyang; LNCT, Liaoning-Changtu; JLCY, Jilin-Chaoyang; JLTJ, Jilin-Taojia; JLLFZ, Jilin-Liufangzi; HLJQA, Heilongjiang-Qinan; HLJSC, Heilongjiang Shuangcheng; HLJBX, Heilongjiang Binxian; HLJHRB, Heilongjiang Harbin. The values without or within the brackets represents fertilizer nitrogen (N) application rate for the optimum nutrient application (OPT) or the farmers’ practice (FP).

Two treatments were set for each experiment consisting of optimum nutrient application (OPT) from Nutrient Expert system and farmers’ practice (FP)^54^. For the OPT treatment, the N fertilizer (urea) application rate ranged from 150 to 211 kg N ha^-1^ at the experimental sites. 1/3 and 2/3 of the total rate was applied as basal fertilizer and side-dressing respectively at the jointing stage at LN and JL sites, whereas 40% and 60% of the total fertilizer was applied as basal and side-dressing respectively at HLJ site. Additionally, the ratio was changed to 1/4, 2/4 and 1/4 at the sowing, jointing and tasselling stages from 2015 to 2016 at JL sites. For the FP treatment, N fertilizer application rate varied from 153 to 280 kg N ha^-1^ at the experimental sites which was 100% applied as basal fertilizer. More detailed management practices for each experiment site are shown in Table 3. The maize grain yield, biomass and N update were measured annually (located within the part of middle four-ridge) at maturity in each treatment which were used for model evaluation. The partial factor productivity of N (PFPN, kg N kg^−1^) was calculated based on grain yield/fertilizer N rate. Detailed measurement information can be referred to Xu et al.^54^.

### DSSAT model

The Decision Support System for Agrotechnology Transfer (DSSAT v4.7, http://dssat.net/) is a mechanistic dynamic model combined with the Crop System Model (CSM), two soil C & N models (the CERES-based and the CENTURY-based soil models) and a soil water balance model (Ritchie method), which simulate crop growth, soil water balance and soil C & N cycling with daily time-step under different cropping systems, management practices and climate conditions^21,45^. The CENTURY-based module was employed to simulate soil C and N processes because it was more suitable for long-term sequence simulations^55^. The CSM-CERES-Maize module was used to simulate maize growth in the experiment years and predict the impacts of climate change scenarios on maize growth. Additionally, the CSM-CROPGRO-Soybean module was used to simulate soybean growth (maize-soybean rotation) under climate change scenarios for management adjustments^23^.

### Model calibration and validation

The DSSAT model requires the following input information: (a) the local daily climate data (e.g., maximum and minimum temperature, precipitation and solar radiation); (b) initial soil condition (e.g., field capacity, wilting point and saturation, soil texture, pH, bulk density and organic carbon content); (c) field management practices (e.g., planting and harvest dates, plant density, tillage, fertilization application rates and times); (d) crop cultivars. The weather data was obtained from the local weather station at the each experimental site. The annual and seasonal mean climate variables from 2011 to 2016 are shown in Supplementary Table S3. The basic soil properties (0–0.20 m soil layer), field management practices and maize cultivar information at each experimental site can be referred to in Table 3.

In this study, the DSSAT model parameters were calibrated using the measurements from OPT treatments for all years at each experimental site (Table 3). Cultivar coefficients must be calibrated to control the crop growth based on local weather, soil conditions and management practices^21^, which mainly include parameters for determine critical phenology stages (P1, thermal time from seedling emergence to the end of the juvenile phase; P2, extent to which development (expressed as days) is delayed for each hour where the photoperiod is greater than 12.5 h and P5, thermal time from silking to physiological maturity), grain filling (G2, maximum possible number of kernels per plant and G3, kernel filling rate during the linear grain filling stage and under optimum conditions), and phylochron interval between successive leaf tip appearances (PHINT). The calibrated cultivar coefficient is listed in Supplementary Table S4. The calibration was conducted by minimizing the root mean square error (RMSE) between the simulated and measured values of maize yield, biomass and N uptake to determine the optimal parameterization. We used a step-wise process to calibrate parameters, and then recalibrate through several steps based on R language until RMSE values were minimized between the simulated and measured values. The datasets from FP treatments were used to validate the model performance. In addition, the DSSAT Sequence program was used to simulate multi-year soil C & N and water dynamics as well as crop growth processes. Initial soil profile data including soil water content and inorganic N (NO_3_^−^–N and NH_4_^+^–N) are only required to be setup before the first year simulation, then soil water, C and N flows could be continuously transferred from the beginning to the end of the simulation automatically^14,23,56^. In this study, the calibrated DSSAT model was then employed to predict the impacts of climate change on maize yields and N use efficiency. The cultivar parameters for soybean were used in the maize-soybean cropping system under future climate change based on the calibration from Liu et al.^23^ In the simulation of maize-soybean rotation, the parameterizing information for rotation maize (e.g., daily climate data, initial soil conditions and field management practices) was consistent with the monoculture maize simulation. The Sequence Analysis mode was used to simulate crop rotations, which could carry-over the soil water and nitrogen processes from one crop to another^57^. Two sequences were created (maize-soybean and soybean-maize rotations) with the same weather conditions to ensure that each phase of the rotation presents in each year. Based on the previous study^23^, on average, soybean was planted on May 1 and harvested on September 30 for each year at all sites, and N fertilizer (urea) was applied at 20 kg N ha^−1^ as basal application.

### Model performance statistics

The model performance statistics can be used to evaluate the difference between simulated and measured data, including root mean square error (RMSE), normalized root mean square error (nRMSE), normalized average relative error (nARE), and index of agreement (d) value (see Eqs. 1–4 below)^58–60^.

1 $${\text{RMSE}} = \sqrt {\mathop \sum \limits_{{{\text{i}} = 1}}^{{\text{n}}} \left( {{\text{S}}_{{\text{i}}} - {\text{M}}_{{\text{i}}} } \right)^{2} /{\text{n}}} { }$$

2 $${\text{nRMSE}} = \left( {\frac{{\sqrt {\mathop \sum \nolimits_{{{\text{i}} = 1}}^{{\text{n}}} \left( {{\text{S}}_{{\text{i}}} - {\text{M}}_{{\text{i}}} } \right)^{2} /{\text{n}}} }}{{{\overline{\text{M}}}}}{ }} \right) \times 100$$

3 $${\text{nARE}} = \left( {\frac{{\frac{1}{n}\mathop \sum \nolimits_{{{\text{i}} = 1}}^{{\text{n}}} \left( {{\text{S}}_{{\text{i}}} - {\text{M}}_{{\text{i}}} } \right)}}{{{\overline{\text{M}}}}}{ }} \right) \times 100$$

4 $${\text{d}} = 1 - \frac{{\mathop \sum \nolimits_{{{\text{i}} = 1}}^{{\text{n}}} \left( {{\text{S}}_{{\text{i}}} - {\text{M}}_{{\text{i}}} } \right)^{2} }}{{\mathop \sum \nolimits_{{{\text{i}} = 1}}^{{\text{n}}} \left( {\left| {{\text{S}}_{{\text{i}}} - {\overline{\text{M}}}} \right| + \left| {{\text{M}}_{{\text{i}}} - {\overline{\text{M}}}} \right|} \right)^{2} }}$$

where S_i_ is the simulated value, M_i_ is the measured value, i = 1, …, n is the number of measured values, and ${\overline{\text{M}}}$ is the mean of the measured values.

For nRMSE, an “excellent”, “good”, “fair” and “poor” model performance is claimed when the nRMSE ≤ 10%, 10 < nRMSE ≤ 20%, 20% < nRMSE ≤ 30% and nRMSE > 30%, respectively^59^. The model performance was satisfactory for yield and biomass if nARE < ± 15%^60^. The value of nARE (%) value indicates underestimation when the nARE < 0 or overestimation when the nARE > 0 compared to the measured values. For d value, d ≥ 0.9, 0.8 ≤ d < 0.9, 0.7 ≤ d < 0.8 and d < 0.7 illustrates “excellent”, “good”, “fair” and “poor” match, respectively^23^.

### Climate change scenarios

In this study, climate change datasets were generated based on the statistical downscaling method^61^. This method relies on empirical relationships between observed climate data and data from global climate models (GCMs). The simulated monthly data by the GCMs were statistically downscaled to the specific sites using the inverse distance-weighted (IDW) interpolation method. A bias correction procedure was applied during this step to correct biases in the site-based monthly GCMs values. And then daily climate variables (maximum and minimum temperature, solar radiation and precipitation) were temporally scaled for each site from the spatially downscaled monthly data through the WGEN stochastic weather generator. Unlike other statistical downscaling methods that require complicated data (e.g., atmospheric circulation or sea surface temperature as predictors) and high computational cost (e.g., dynamical downscaling), statistical downscaling method is reliable and rapid mainly due to the use of historical observation data to modify the monthly GCMs data and low computational cost. These climate data obtained from this method could match with crop models in order to explore the future climate impacts on agricultural systems. Due to the complexity of the climate system, the future climate data was produced by Climate System Models from Beijing Climate Centre BCC-CSM1.1 (BC1) and BCC-CSM1.1 (m) (BC2) which were obtained from the Coupled Model Inter-comparison Project phase 5 (CMIP5) to drive the DSSAT model in this study. The BC1 model is a fully coupled global climate-carbon model with an interactive vegetation and global carbon cycle, which includes the atmospheric component BCC Atmospheric General Model version 2.1 (BCC_AGCM2.1) with a horizontal resolution of T42 (approximately 2.8125° × 2.8125° transformed grid), 26 levels in a hybrid sigma/pressure vertical coordinate system with the top level at 2.914 hPa, ocean component Modular Ocean Model version 4 (MOM4)-L40, land component BCC Atmosphere and Vegetation Interaction Model version 1.0 (BCC_AVIM1.0), and sea ice component [sea ice simulator (SIS)]^62^. The BC2 model is established to increase the horizontal resolution of the atmospheric component and land component to T106 (approximately 1.125° × 1.125° transformed grid), forming BCC_AGCM2.2 and BCC_AVIM1.1, respectively based on the BC1 version^63^. Compared to the BC1 model, the BC2 model with finer horizontal resolution performed better for some climate characteristics. For example, the spatial variability of the simulated climatological monthly precipitation is closer to the observations and tropical sea surface temperature annual cycles in the tropical ocean are generally more reasonable^63^. In this study, both climate models (BC1 and BC2) were used to explore climate change impacts on maize production in Northeast China.

Observed daily meteorological data at all sites during 1980 to 2010 as baseline were obtained from the Chinese Meteorological Data Service Centre (CMDC, http://data.cma.cn/en). Two Representative Concentration Pathway scenarios (RCP4.5, intermediate scenario and RCP 8.5, high emissions scenario) based upon radiative forcing levels of 4.5 and 8.5 w/m^2^ at the end of the 21st century, respectively were selected for two future periods from the 2050s (2041–2070) and the 2080s (2071–2100). Historical meteorological data from one representative experiment in each province is selected to be used for downscaling and bias-correction of the future climate data from the GCMs. The calibrated parameters for maize cultivars of FY9, NH101 and GF1 at LN, JL and HLJ sites respectively were used in the climate scenario simulations (Supplementary Table S3). The comparison of historical and projected climate variables at LN, JL and HLJ sites is shown in Supplementary Table S2.

### Adaptation strategies

Four adaptation strategies were explored to mitigate the negative impacts of climate change on maize production based on sensitivity analysis under baseline and future climate scenarios in Northeast China. The management practice scenarios include the following: (a) fertilizer N application rates were simulated from 0 to 300 kg N ha^−1^ with a 30 kg N ha^−1^ interval and split fertilizer application was simulated as basal (1/3 of the total) and sidedressing (2/3 of the total at the jointing stage)^26,37,54^; (b) Maize-soybean rotation was considered compared to the maize monoculture^23^; (c) Planting dates were shifted early/late with a 10 day interval based on the default (123 day at LN and JL, 128 day at HLJ)^26,14^; (d) Maize cultivar with longer growing seasons were developed based on two thermal time parameters of P1 [thermal time from seedling emergence to the end of the juvenile phase (degree days > 8 °C)] ranging from 160 to 425 °C.d. with a 50, 30 and 25 °C.d. interval and P5 (thermal time from silking to physiological maturity, (degree days > 8 °C) ranging from 656 to 980 °C.d. with a 50 °C.d. interval at LN, JL and HLJ, respectively^14^. For default parameters, planting date (123, 123 and 128 day) and fertilizer application rate (210, 210 and 180 kg N ha^−1^) as basal at LN, JL and HLJ sites will be used in the future climate scenarios, respectively.

## Supplementary information

Supplementary Information.
