# Supplementary material for: Modelling adaptation strategies to reduce adverse impacts of climate change on maize cropping system in Northeast China
Source: Sci Rep. 2021 Jan 12;11:810. doi: 10.1038/s41598-020-79988-3 (PMC7804944; doi:10.1038/s41598-020-79988-3)
Supplement: Supplementary file 1 — Supplementary Information. [file 41598_2020_79988_MOESM1_ESM.pdf]

Supplementary Information

Modelling adaptation strategies to reduce adverse impacts of climate change on maize cropping system in Northeast China

Rong Jiang [1, 2], Wentian He [3, *], Liang He [4], J.Y. Yang [2], B. Qian [5], Wei Zhou [1], Ping He [1, *]

[1] *Ministry of Agriculture Key Laboratory of Plant Nutrition and Fertilizer, Institute of Agricultural Resources and Regional Planning, Chinese Academy of Agricultural Sciences (CAAS), Beijing 100081, China*

[2] *Harrow Research and Development Centre, Agriculture & Agri-Food Canada, 2585 County Road, Harrow, Ontario N0R 1G0, Canada*

[3] *Institute of Plant Nutrition and Resources, Beijing Academy of Agriculture and Forestry Sciences, Beijing 100097, China*

[4] *National Meteorological Centre, Beijing 100081, China*

[5] *Ottawa Research and Development Centre, Agriculture & Agri-Food Canada, 960 Carling Ave, Ottawa, Ontario K1A 0C6, Canada*

∗ Corresponding authors.

E-mail addresses: wentian_he@hotmail.com, heping02@caas.cn

Site	Management	Item	Optimized	PFPN (kg N kg^{-1})				
				Baseline	RCP 4.5 2050s	RCP 4.5 2080s	RCP 8.5 2050s	RCP 8.5 2080s
LN	Default	-	-	37.2	35.7	33.5	27.7	23.4
	N rate (monoculture)	Base	240	33.5	32.4	30.5	24.7	21.1
	(kg N ha^{-1})	Base and side-dress	210 (1/3 and 2/3)	38.2	36.9	34.9	28.3	24.2
	N rate (rotation) (kg N ha^{-1})	Base	210	41.7	40.7	38.9	31.1	28.1
	Planting date (day of year)	Baseline/Future	133/143	46.6	47.5	44.1	37.7	33.9
	Cultivar parameters (°C.d.)	P1	325-375	44.5	43.0	41.7	35.8	28.9
		P5	980	47.9	47.6	45.3	37.7	31.8
JL	Default	-	-	51.1	44.6	40.2	39.1	33.7
	N rate (monoculture)	Base	240	45.1	39.9	36.6	35.3	29.7
	(kg N ha^{-1})	Base and side-dress	210 (1/3 and 2/3)	51.6	45.3	42.0	40.7	33.9
	N rate (rotation) (kg N ha^{-1})	Base	210	52.7	48.6	43.9	42.8	36.7
	Planting date (day of year)	Baseline/Future	113/143	59.7	52.8	51.2	49.3	44.2
	Cultivar parameters (°C.d.)	P1	260-290	59.6	53.9	50.5	48.8	41.1
		P5	920	64.6	61.6	55.8	54.5	46.5
HLJ	Default	-	-	47.0	44.2	43.0	39.5	36.2
	N rate (monoculture)	Base	210	40.7	38.0	36.9	34.3	31.7
	(kg N ha^{-1})	Base and side-dress	180 (1/3 and 2/3)	47.5	44.6	43.4	40.2	37.4
	N rate (rotation) (kg N ha^{-1})	Base	180	51.4	47.4	46.2	43.5	39.2
	Planting date (day of year)	Baseline/Future	138/148	49.5	52.8	50.8	47.9	43.1
	Cultivar parameters (°C.d.)	P1	210-235	47.0	46.5	45.4	42.6	39.3
		P5	856	52.2	55.3	54.8	49.4	46.2

Table S1. Potential adaptation management practices for partial factor productivity of nitrogen (PFPN) under climate change scenarios in Liaoning (LN), Jilin (JL) and Heilongjiang (HLJ) provinces in Northeast China. P1, Thermal time from seedling emergence to the end of the juvenile phase (degree days > 8 °C); P5, Thermal time from silking to physiological maturity (degree days > 8 °C).

Location	Climate model	Scenario	Period	Annual					Seasonal (May-September)				
				Tmax (°C)	Tmin (°C)	Tavg (°C)	Precipitation (mm)	SRAD (MJ m^{-2} d^{-1})	Tmax (°C)	Tmin (°C)	Tavg (°C)	Precipitation (mm)	SRAD (MJ m^{-2} d^{-1})
LN	BC1	Baseline	1980-2010	16.4	2.9	9.6	489	14.4	28.1	15.7	21.9	428	18.6
		RCP4.5	2041-2070	17.9	5.1	11.5	663	14.3	29.6	17.8	23.7	591	18.5
		RCP4.5	2071-2100	18.2	5.8	12.1	636	14.5	28.7	18.2	24.1	557	18.8
		RCP8.5	2041-2070	18.8	6.1	12.5	529	14.5	30.6	18.8	24.7	453	18.9
		RCP8.5	2071-2100	20.4	8.4	14.4	626	14.5	31.8	20.5	26.2	550	18.9
	BC2	Baseline	1980-2010	16.0	2.5	9.3	517	14.3	27.7	15.3	21.5	454	18.4
		RCP4.5	2041-2070	17.2	4.0	10.6	591	14.4	28.7	16.3	22.5	514	18.7
		RCP4.5	2071-2100	17.5	4.6	11.0	608	14.5	28.9	16.7	22.8	528	18.8
		RCP8.5	2041-2070	18.0	5.1	11.6	566	14.5	29.3	17.3	23.3	506	18.8
		RCP4.5	2071-2100	18.9	6.6	12.7	644	14.4	30.2	18.5	24.3	559	18.9
JL	BC1	Baseline	1980-2010	11.4	0.6	6.0	614	13.2	24.9	14.3	19.6	529	17.6
		RCP4.5	2041-2070	12.8	2.3	7.5	670	13.3	26.1	15.8	21.0	571	17.9
		RCP4.5	2071-2100	12.9	2.9	8.0	668	13.3	24.8	16.1	21.2	568	17.9
		RCP8.5	2041-2070	13.5	3.2	8.3	679	13.4	26.8	16.7	21.8	578	18.0
		RCP8.5	2071-2100	15.0	5.1	10.1	677	13.4	27.9	18.1	23.0	581	18.0
	BC2	Baseline	1980-2010	11.3	0.5	5.9	586	13.1	24.7	14.1	19.4	503	17.4
		RCP4.5	2041-2070	12.5	1.9	7.2	663	13.2	25.5	15.0	20.2	571	17.7
		RCP4.5	2071-2100	13.0	2.6	7.8	653	13.3	25.8	15.3	20.6	558	17.8
		RCP8.5	2041-2070	13.3	2.8	8.1	622	13.2	26.2	15.8	21.0	535	17.8
		RCP8.5	2071-2100	14.5	4.3	9.4	756	13.1	27.0	16.6	21.8	648	18.0
HLJ	BC1	Baseline	1980-2010	10.3	-1.3	4.5	523	13.4	24.7	13.4	17.1	453	18.8
		RCP4.5	2041-2070	11.8	0.5	5.3	563	13.4	26.1	14.8	18.5	472	19.1
		RCP4.5	2071-2100	12.1	0.9	5.9	593	13.4	26.1	14.9	18.6	500	18.9
		RCP8.5	2041-2070	12.5	1.2	6.1	598	13.4	26.7	15.5	19.1	501	19.0
		RCP8.5	2071-2100	13.9	3.0	7.8	597	13.3	27.8	16.8	20.3	502	19.1
	BC2	Baseline	1980-2010	10.2	-1.4	6.1	534	13.4	24.5	13.1	13.8	456	18.6
		RCP4.5	2041-2070	11.4	0.1	4.9	567	13.4	25.5	14.0	17.7	478	19.0
		RCP4.5	2071-2100	11.9	0.7	5.6	576	13.4	25.8	14.3	18.1	497	19.0
		RCP8.5	2041-2070	12.3	0.9	5.9	513	13.5	26.2	14.8	18.5	433	19.2
		RCP8.5	2071-2100	13.5	2.1	7.1	673	13.3	26.9	15.4	19.1	561	19.0

Table S2. Changes in annual and seasonal mean maximum and minimum temperature, precipitation and solar radiation under different climate change scenarios at Liaoning (LN), Jilin (JL) and Heilongjiang (HLJ) provinces in Northeast China. Tmax, maximum temperature; Tmin, minimum temperature; SRAD, solar radiation; Tavg, average temperature. BC1, BCC-CSM1.1 climate model; BC2, BCC-CSM1.1 (m) climate model.

Location	Maximum temperature (°C)		Minimum temperature (°C)		Precipitation (mm)		Solar radiation (MJ m^{-2} d^{-1})	
	Annual	Seasonal	Annual	Seasonal	Annual	Seasonal	Annual	Seasonal
LNCY	15.2	27.1	2.8	15.8	523	432	14.8	18.9
LNCT	11.9	25.5	2.9	17.3	635	492	14.9	19.8
HLJQA	8.5	24.0	-2.2	14.1	619	520	13.7	18.5
HLJSC	10.2	24.9	-0.5	14.3	625	536	13.5	18.1
HLJBX	9.8	24.6	-1.0	13.9	610	497	13.9	19.3
HLJHRB	10.3	25.2	0.0	15.2	546	460	12.4	17.1
JL	11.7	25.6	1.6	15.4	648	527	14.4	20.2

Table S3. Annual and seasonal mean maximum and minimum temperature, precipitation and solar radiation from 2011 to 2016 at Liaoning (LN), Jilin (JL) and Heilongjiang (HLJ) provinces in Northeast China. Seasonal, May to September. LNCY, Liaoning-Chaoyang; LNCT, Liaoning-Changtu; HLJQA, Heilongjiang-Qinan; HLJSC, Heilongjiang Shuangcheng; HLJBX, Heilongjiang Binxian; HLJHRB, Heilongjiang Harbin.

Parameter	Description	Calibrated cultivar coefficient								
		FY9	TY120	YH33	ZF62	NH101	MJN205	LM33	HN1	GF1
P1	Thermal time from seedling emergence to the end of the juvenile phase (degree days > 8 °C)	325	265	260	260	260	210	210	205	210
P2	Extent to which development (expressed as days) is delayed for each hour increase in photoperiod > the longest photoperiod 12.5 hours	0.5	0.5	0.5	0.5	0.5	0.5	0.5	0.5	0.5
P5	Thermal time from silking to physiological maturity (degree days > 8 °C)	880	945	868	800	820	728	810	762	756
G2	Maximum possible number of kernels per plant	780	820	682	746	765	697	600	647	680
G3	Kernel filling rate during the linear grain filling stage and under optimum conditions (mg/day)	8.60	9.48	7.8	7.08	7.65	6.89	6.80	6.21	7.00
PHINT	Phylochron interval between successive leaf tip appearances (degree days)	38.9	36.9	48.9	45.9	42.9	42.9	42.9	47.9	38.9

Table S4. The calibrated cultivar coefficients for maize at Liaoning (LN), Jilin (JL) and Heilongjiang (HLJ) provinces in Northeast China.

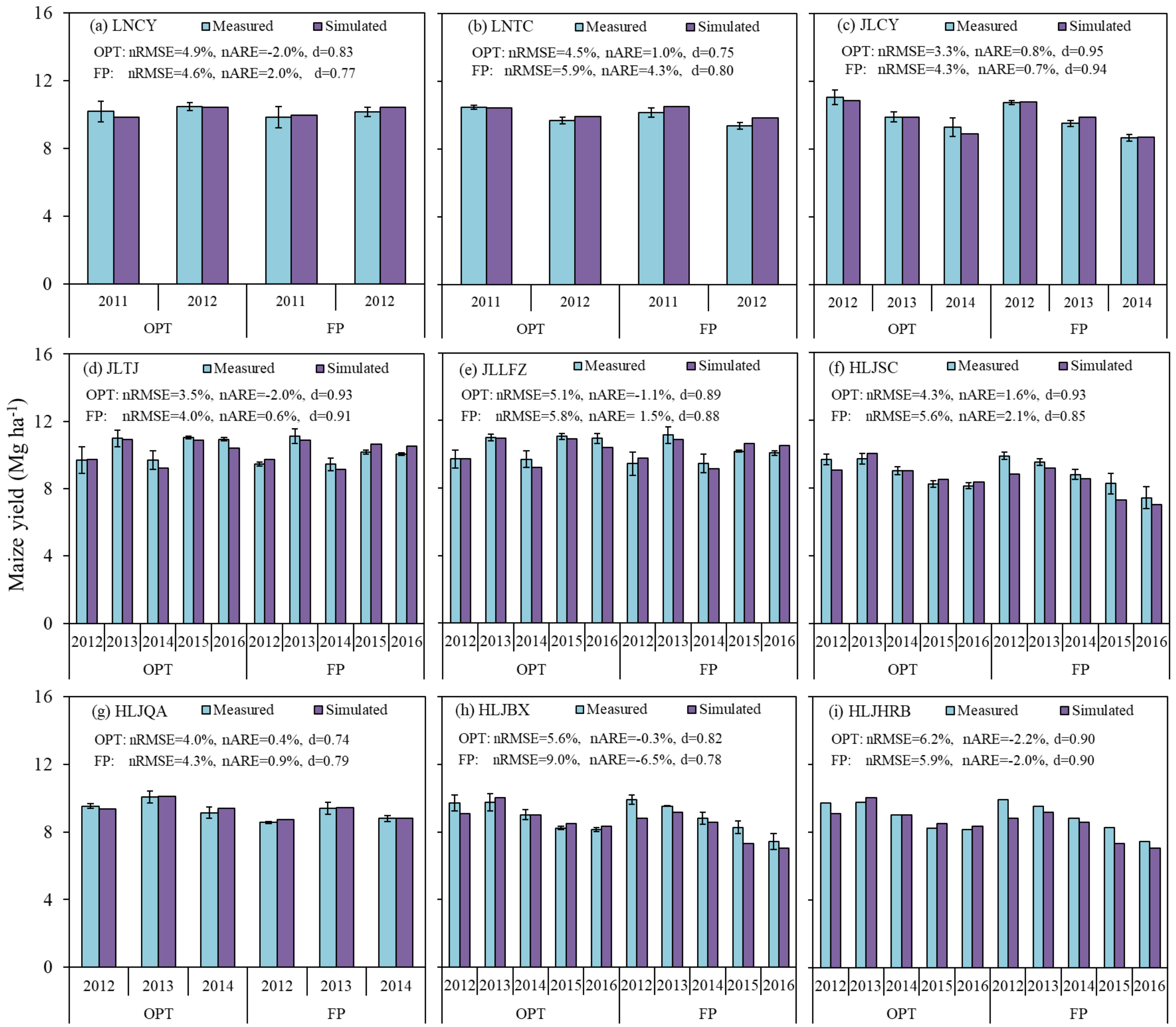

Figure S1. Measured and simulated maize yields from 2011 to 2016 for the optimum nutrient application (OPT) and the farmers' practice (FP) at LNCY (a), LNCT (b), JLCY (c), JLTJ (d), JLLFZ (e), HLJSC (f), HLJQA (g), HLJBX (h) and HLJHRB (i) in Northeast China.

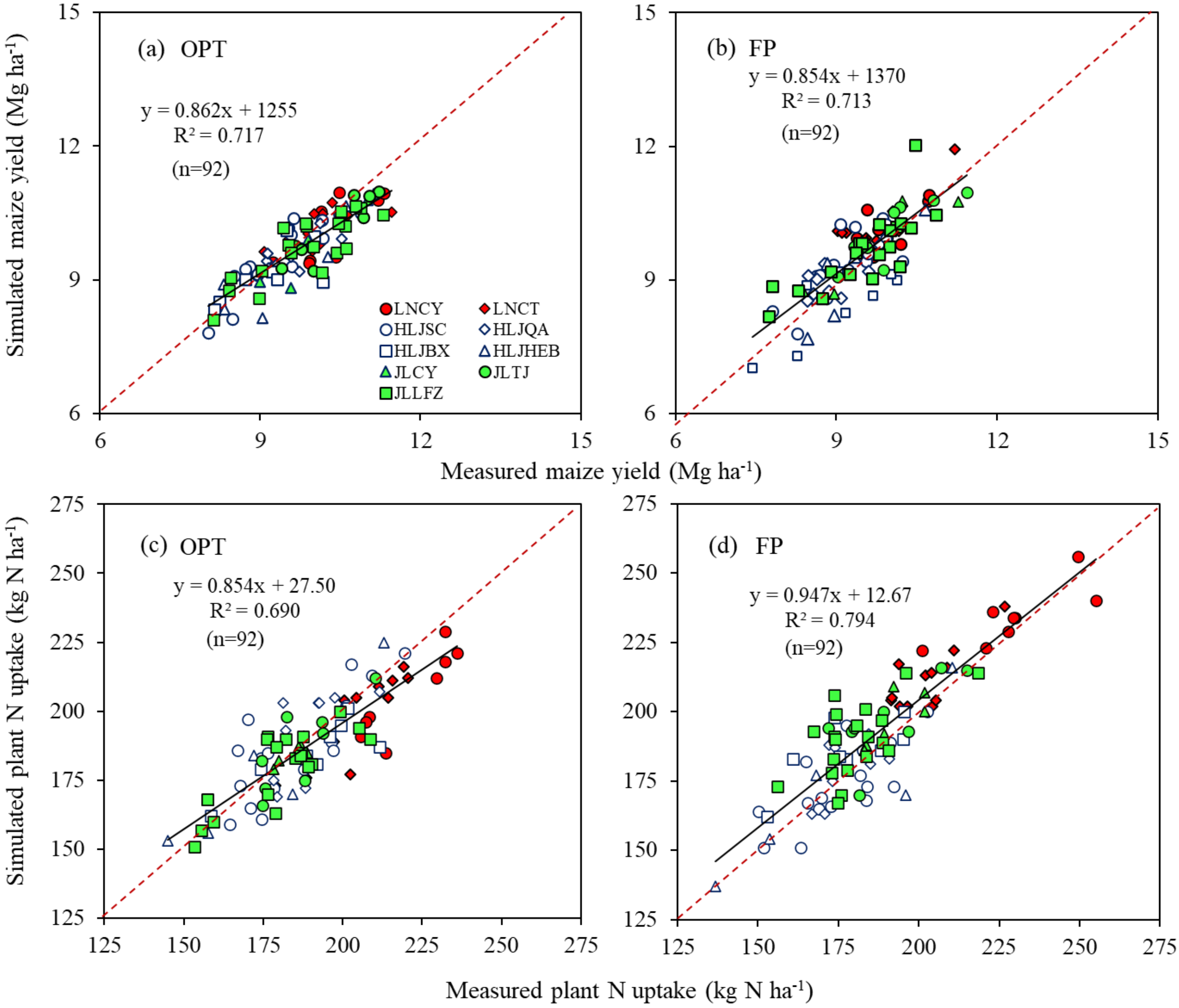

Figure S2. Measured and simulated maize yields and plant nitrogen (N) uptake from 2011 to 2016 for the optimum nutrient application (OPT) (a, c) and the farmers' practice (FP) (b, d) in Northeast China.

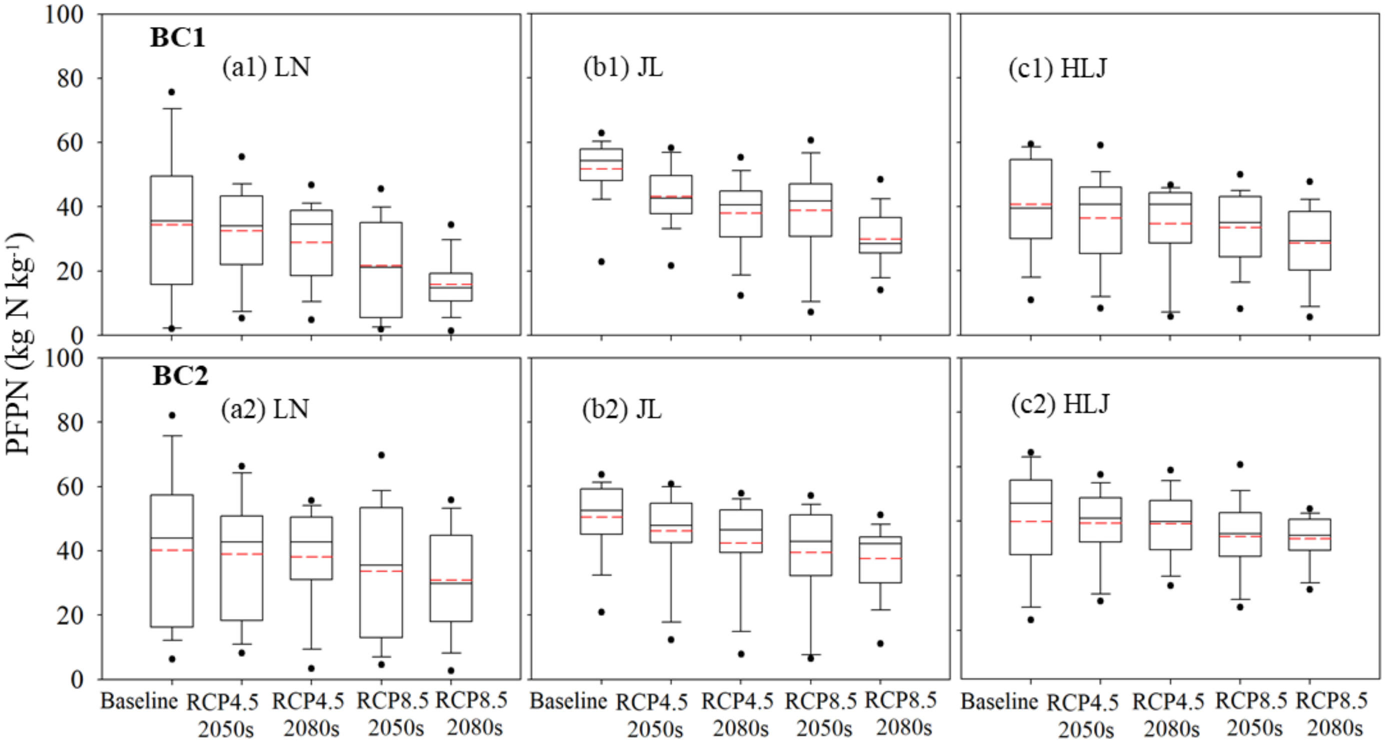

Figure S3. Effects of climate change scenarios on partial factor productivity of nitrogen (PFPN) under BC1 (a1, b1, c1) and BC2 (a2, b2, c2) climate scenarios at Liaoning (LN), Jilin (JL) and Heilongjiang (HLJ) provinces in Northeast China. The black (solid) and red (dashed) lines, lower and upper edges of the boxes, and bars and dots outside the boxes represent median and mean values, 25th and 75th, 5th and 95th, and <5th and >95th percentiles of all data, respectively.

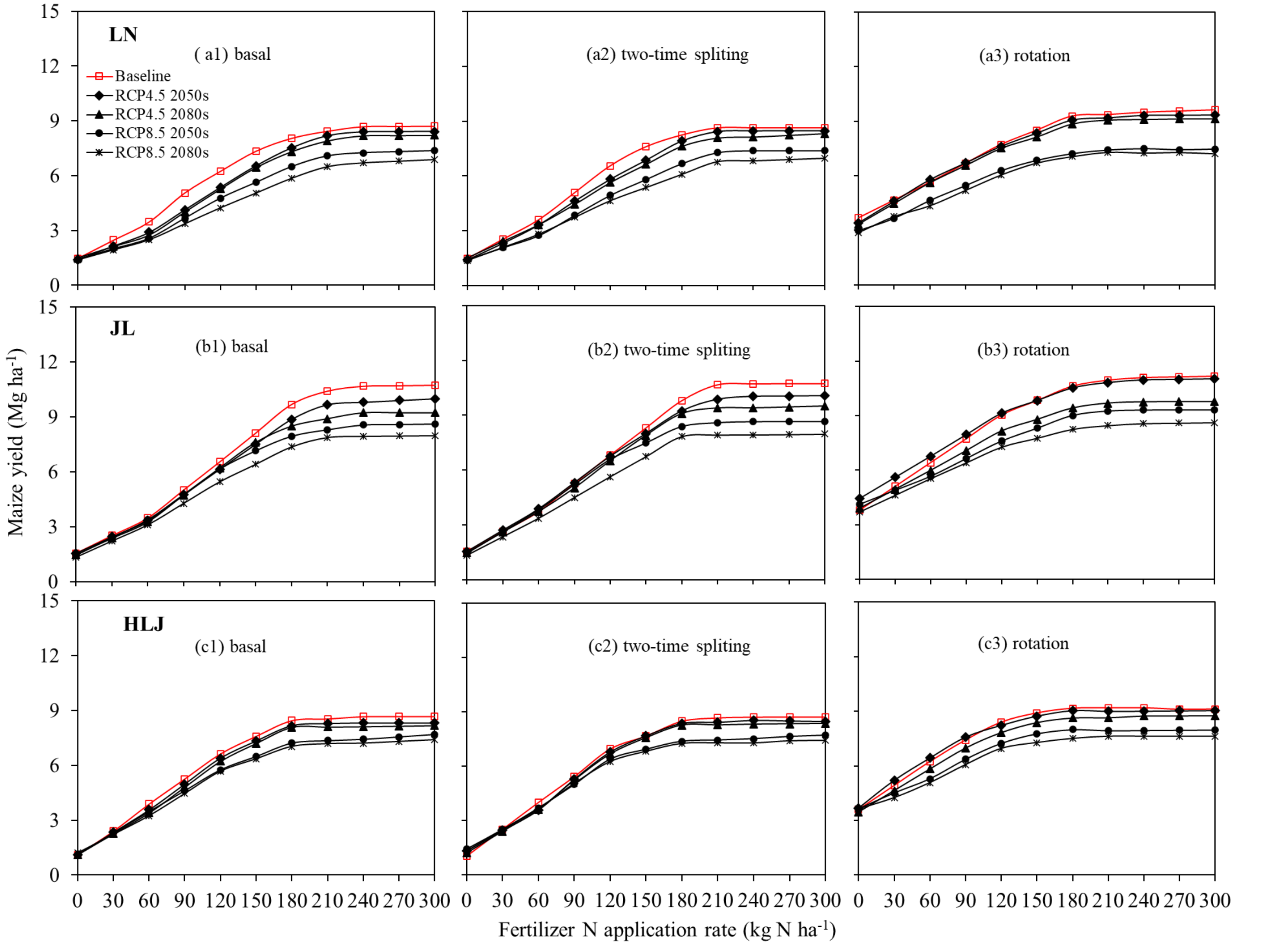

Figure S4. Responses of maize yields to nitrogen (N) application rate as basal (a1, b1, c1), as two-time splitting (a2, b2, c2) under maize monoculture and as basal under maize-soybean rotation (a3, b3, c3) under climate change scenario BC2 (BCC-CSM1.1 (m)) at Liaoning (LN), Jilin (JL) and Heilongjiang (HLJ) provinces in Northeast China.
